# Importance of a registered and structured protocol when conducting systematic reviews: comments about nebulized antibiotics for ventilator-associated pneumonia

**DOI:** 10.1186/s13054-015-1020-8

**Published:** 2015-08-20

**Authors:** Fernando G. Zampieri, Antonio P. Nassar, Dimitri Gusmao-Flores, Leandro U. Taniguchi, Antoni Torres, Otavio T. Ranzani

**Affiliations:** Cooperative Network for Research—AMIB-Net, Associação de Medicina Intensiva Brasileira, Rua Arminda, 93, 7 andar, São Paulo, 04545-100 Brazil; Emergency Medicine Discipline, Faculty of Medicine, University of São Paulo, Rua Dr. Enéas de Carvalho Aguiar, 255, 5th floor, room 5023, São Paulo, 05403-010 Brazil; Intensive Care Unit, Hospital Alemão Oswaldo Cruz, Rua João Julião, 331, São Paulo, 01323-903 Brazil; Adult Intensive Care Unit, A.C. Camargo Cancer Center, Rua Professor Antônio Prudente, 211, São Paulo, 01509-010 Brazil; Intensive Care Unit, University Hospital Prof. Edgar Santos, Universidade Federal da Bahia, Rua Augusto Viana, Salvador, 40110-910 Brazil; Research and Education Institute (IEP), Hospital Sirio-Libanes, Rua Prof. Daher Cutait, 69, São Paulo, 01308-060 Brazil; Department of Pulmonology, Hospital Clinic of Barcelona, Institut D’investigacions August Pi I Sunyer (IDIBAPS), University of Barcelona, Ciber de Enfermedades Respiratorias (CIBERES), Carrer Villarroel, 170, Barcelona, 08036 Spain; Amil Critical Care Group, Hospital Paulistano, Rua Martiniano de Carvalho, 741, São Paulo, 01321-001 Brazil; Respiratory Intensive Care Unit, Pulmonary Division, Heart Institute, Hospital das Clínicas, University of São Paulo, Av. Dr. Arnaldo, 455 Laboratório de Pneumologia, 2° andar, sala 2144, Cerqueira César, 01246903 Sao Paulo, Brazil

We appreciate Gu’s [1] interest in our study. We apologize and agree with his comment about attributing units to standardized mean difference (SMD). Nevertheless, similar to the SMD, results in mean difference (control – nebulized) were unaffected by nebulized antibiotics (2.67 days, 95 % confidence interval (CI) –2.89, 8.23 for ICU length of stay (LOS); and 0.70 days, 95 % CI −3.40, 4.80 for mechanical ventilation). However, we strongly disagree with other points raised by the letter.

First, the study protocol was defined a priori [2]. We disagree that combining observational studies with intervention studies is reserved only for safety evaluation. This topic has been discussed in the literature and combining both types of studies was adequate for our aim [3]. Furthermore, we presented the main results separating interventional studies from observational studies, thereby allowing the reader to interpret both analyses independently.

Second, both of the studies cited as “case–control studies” [1] received this denomination in their title and abstract. However, by reading their methods it becomes clear that they are actually matched cohort studies [4, 5]. Indeed, they matched exposed patients (“nebulized group”) to unexposed patients (“no-nebulized group”). A case–control design starts with the outcome (case = “clinical success”) and matches them with controls (“clinical failures”). Therefore, our measure of effect was correct [5]. For exploration, we report the analysis for clinical cure using the odds ratio (OR) (Fig. 1). The results are unchanged.

**Fig. 1:**
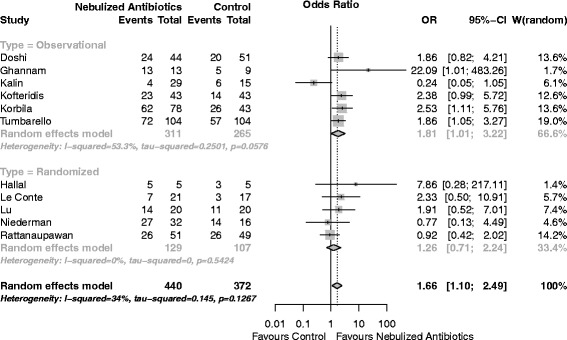
Forest plot for clinical cure using odds ratios (OR). *P* for overall effect = 0.015. *CI* confidence interval

Third, Kalin’s study was included because it fulfilled our inclusion/exclusion criteria [2]. Gu’s suggestion to exclude this study based solely on its effects in heterogeneity could be considered selective reporting [1].

Our study provided data for further trials aiming to evaluate the effect of nebulized antibiotics in ventilator-associated pneumonia (VAP) [2].

## Abbreviations

CI: Confidence interval
LOS: Length of stay
OR: Odds ratio
SMD: Standardized mean difference
VAP: Ventilator-associated pneumonia
